# The Association of Health Insurance with institutional delivery and access to skilled birth attendants: evidence from the Kenya Demographic and health survey 2008–09

**DOI:** 10.1186/s12913-017-2397-7

**Published:** 2017-07-03

**Authors:** Lawrence P.O. Were, Edwin Were, Richard Wamai, Joseph Hogan, Omar Galarraga

**Affiliations:** 1 Department of Health Sciences, Boston University’s College of Health and Rehabilitation Sciences: Sargent College, Boston, USA; 2 School of Public Health, Brown University, Providence, RI USA; 3 Department of Reproductive Health, Moi University & AMPATH-Kenya, Eldoret, Kenya; 4 Department of Cultures, Societies and Global Studies, Northeastern University, Boston, MA USA

**Keywords:** Healthcare financing, Insurance, Institutional delivery, Skilled birth attendants, Socio-economic status

## Abstract

**Background:**

Healthcare financing through health insurance is gaining traction as developing countries strive to achieve universal health coverage and address the limited access to critical health services for specific populations including pregnant women and their children. However, these reforms are taking place despite limited evaluation of impact of health insurance on maternal health in developing countries including Kenya. In this study we evaluate the association of health insurance with access and utilization of obstetric delivery health services for pregnant women in Kenya.

**Methods:**

Nationally representative data from the Kenya Demographic and Health Survey 2008–09 was used in this study. 4082 pregnant women with outcomes of interest - Institutional delivery (Yes/No – delivery at hospital, dispensary, maternity home, and clinic) and access to skilled birth attendants (help by a nurse, doctor, or trained midwife at delivery) were selected from 8444 women ages 15–49 years. Linear and logistic regression, and propensity score adjustment are used to estimate the causal association of enrollment in insurance on obstetric health outcomes.

**Results:**

Mothers with insurance are 23 percentage points (*p* < 0.01) more likely to deliver at an institution and 20 percentages points (*p* < 0.01) more likely have access to skilled birth attendants compared to those not insured. In addition mothers of lower socio-economic status benefit more from enrollment in insurance compared to mothers of higher socio-economic status. For both institutional delivery and access to skilled birth attendants, the average difference of the association of insurance enrollment compared to not enrolling for those of low SES is 23 percentage points (*p* < 0.01), and 6 percentage points (*p* < 0.01) for those of higher SES.

**Conclusions:**

Enrolling in health insurance is associated with increased access and utilization of obstetric delivery health services for pregnant women. Notably, those of lower socio-economic status seem to benefit the most from enrollment in insurance.

**Electronic supplementary material:**

The online version of this article (doi:10.1186/s12913-017-2397-7) contains supplementary material, which is available to authorized users.

## Background

Healthcare financing through health insurance is receiving considerable attention in developing countries while generating substantial debate globally as the third health revolution [1]. Mechanisms such as national health insurance [2–5], community based health insurance [6, 7], and targeted public health insurance [8, 9] are being implemented in several countries. The backdrop is the World Health Organization’s (WHO) global agenda on Universal Health Coverage (UHC), which calls for access of all people to comprehensive health services at affordable costs and without financial hardship through protection against catastrophic health expenditures [10]. At the center of this agenda is increased access to healthcare for households that face health shocks and that rely on out-of-pocket (OOP) payments [11]. In addition, countries continue to contend with donor dependency and loss of personnel and productivity of their healthcare workforce [12–15]. In response, health systems in developing countries are undergoing reforms, seeking ways to enhance their healthcare financing mechanisms, to effectively and efficiently manage their human resources for health and improve the health of their citizens [15, 16]. The WHO agenda also highlights the importance of creating an evidence base for healthcare financing reforms [17].

These healthcare financing reforms are especially important for maternal and child health. According to the 2013 Millennium Development Goals (MDG) Report, most maternal deaths are preventable but progress in this area is not optimal. Globally, maternal mortality ratio declined by 43.9% between 1990 and 2015 from 385 to 216 maternal deaths per 100,000 live births [18]. This progress however, fell far short of the MDG target of reducing maternal mortality by 75% by 2015 [19]. Also, in sub-Saharan Africa, one in nine children die before age five accounting for a growing share of child deaths [18]. This is more than 16 times the average mortality for newborns in their first month of life in developed countries [19]. The MDG report notes that meeting the targets of reducing child mortality and improving maternal health require accelerated interventions, including improved access to emergency interventions, such as improved access to emergency obstetric care, assistance from skilled health personnel at delivery and the provision of antiretroviral therapy to all pregnant women who need it [19]. However, lack of adequate healthcare financing including health insurance is associated with limited access to these critical interventions even in developed countries [20]. Thus the ongoing healthcare financing reforms in general and specific to maternal and child health are critical and timely.

Specific to maternal and obstetric health in Kenya, the 2014 Demographic and Health Survey (DHS) shows that the Maternal Mortality Rate (MMR) was 362 per 100,000 live births with 61% of births in health facilities and 39% at home [21]. And in 2008–09 the DHS showed that the MMR was 488 maternal deaths per 100,000 live births and 56% of Kenyan women gave birth at home and 44% in health facilities [22]. This makes Kenya among the 10 most dangerous countries for pregnant women [23] despite the tremendous progress the country has made in the last decade. In addition, Kenya is ranked the 39th country with the highest under-5 mortality [22], and in 2009, Kenya experienced over 34,000 stillbirths [24]. The ministry of health thus recommends that every pregnant woman receive skilled care during delivery [25]. In addition, The 2010 Constitution of Kenya provides a legal framework to ensure a comprehensive rights based delivery of health services while the Kenya Health Policy Framework, first released in 1994 and revised for 2012–2030, seeks to provide quality health care that is ‘affordable, equitable, accessible and responsive to all’ [26, 27]. These policy documents consider health insurance to be a progressive way of financing healthcare access.

Nevertheless, enrollment in health insurance in Kenya is low. In 2008–09, 7% of women and 11% of men aged 15–49 years had health insurance coverage [22]. As of 2014, there were six (6) types of insurance coverage available including the National Insurance Scheme - National Hospital Insurance Fund (NHIF), Employer Based Insurance, Mutual Health Organization/Community Based Insurance, Privately Purchased Insurance, Prepayment Scheme, among others that covered 18% of women and 21% of men [28]. While there is an increase from 2008 to 09, the 2014 data shows that 82% of women and 79% of men are still without insurance coverage.

Moreover, the government has on numerous occasions waived user fees for delivery related services to ensure that pregnant women are able to access institutional delivery and receive skilled care during delivery. However, the Kenya Service Provision and Assessment (SPA) 2004 and 2010 shows that 80% of facilities offering delivery related services charge user fees for these services [29]. According to the report, user fees are most common in faith based organizations (99%), private institutions (97%) and government facilities (74%) but are uncommon in non-governmental organization (NGO) facilities (19%) – Additional file 1: Figures A1 and .2 in the appendix graph the charging of user fees for delivery services.

In addition, a review of the literature specific to Kenya shows that many of the studies look at the determinants of enrollment in insurance, utilization of maternal health, and the economic burden of maternal health. Three studies utilizing the 2008–09 DHS data look at: the determinants of health insurance ownership among women [30], the factors influencing place of delivery for women in Kenya [31], and the individual and contextual determinants of adequate maternal health care services [32]. Another study looks at the economic burden of maternal mortality on households in rural western Kenya [33]. This lack of studies looking at the impact of health insurance on health outcomes is despite Kenya having the oldest Social Health Insurance (SHI) - the National Hospital Insurance Fund (NHIF) – in sub-Saharan Africa established in 1966 [34]. An operational review of the NHIF was recently conducted by the World Bank/International Finance Cooperation (IFC) [35]. The review showed that NHIF faced various challenges in its reform efforts and for it to act as the principal financier in a universal health insurance scheme in Kenya, it needed to improve its transparency and financial management practices.

Given this confluence of ongoing health and healthcare financing reforms, high maternal and child morbidity and mortality, user fee charges for institutional delivery, and the availability of a Social Health Insurance scheme, it is important to establish the impact of health insurance on maternal health outcomes in Kenya. Our study hypothesizes that insurance improves access and utilization of healthcare services, and improves maternal and child health. This hypothesis is based on economic theory suggesting that people purchase health insurance not only to avoid risk of financial loss, but also as a mechanism for gaining access to healthcare that would otherwise be unaffordable [36, 37]. Such an evaluation would not only be important for policy dispensation in Kenya but also in countries in similar settings in the global South working on healthcare financing reforms.

## Methods

### Data and variables

We used data from the 2008–09 Kenya Demographic and Health Survey (KDHS) for this analysis. KDHS is a nationally representative survey that sampled 10,000 households [22] and collected detailed health and sociodemographic information. A total of 400 clusters—133 urban and 267 rural—were selected from the master frame [22]. This sample was constructed to allow for separate estimates for key indicators for each of the eight provinces in Kenya, as well as for urban and rural areas separately [22]. Urban areas were oversampled to get enough cases for analysis [22]. As a result the KDHS sample is not self-weighting at the national level; therefore, the empirical strategies implemented in this analysis are based on weighted data.

The women’s sample comprises of 8444 women ages 15–49. The final analytic sample has 4082 women who report two outcomes of interest – institutional delivery (Yes/No – delivery at hospital, dispensary, maternity home, and clinic) and access to skilled birth attendants (help by a nurse, doctor, or trained midwife at delivery). This definition of skilled birth attendant is based on the WHO recommendations [38]. The final analytic dataset of 4082 takes into account three (3) mothers who were missing values on their outcomes as well as covariates. Given that this represents 0.04% missing values, the missingness was ignored and the three mothers were dropped from the analysis.

The independent variable is insurance enrollment (Yes/No). We generated the variable by combining responses to enrollment in different kinds of health insurance – community based health insurance, insurance from employer, government or social security, privately purchased insurance and insurance from other source. The 2008–09 KDHS was the first survey to include questions on insurance enrollment. Insurance enrollment however, is not random as individuals can select whether or not to enroll in insurance and at what time during the year they actually enroll.

Because insurance enrollment is not a random event, we use a selection of covariates in the analysis including age, marital status, education, total number of children, total number of household members, employment status, urban or rural residence, HIV test, frequency of reading newspapers, cooking fuel and whether or not they have electricity. This vector of covariates allows for appropriate regression adjustment and use of the observed characteristics to construct counterfactuals of enrollment in insurance based on propensity scores.

### Empirical strategy

We would require a counterfactual to estimate the causal effects of insurance status on access to care for pregnant women [39] -- i.e., what would have happened to the women in the absence of the intervention - in this case enrollment in insurance. The ideal way of achieving a counterfactual is through randomization. However, insurance enrollment is not randomized thus this observational study uses rigorous non-experimental methods.

First, we estimate the association between health insurance and healthcare access using unadjusted and adjusted linear and logistic models. In the logistic regressions, we estimated the marginal effects – Table 3. We estimated models of the general form:

1 $$ \mathit{\Pr}\left({y}_i=1\right)= logit-1\ \left({X}_i\beta \right) $$

where: the subscript *i* runs over observations *i* = 1 , … , *n*; *y*_*i*_ is the outcome of interest (institutional delivery or skilled birth attendant); *Xβ* is the linear predictor.

However, as selection into insurance is not random and in order to make any empirical estimations of the causal association with insurance, the adverse selection has to be accounted for. To reduce selection on observables, we implemented propensity score methods based on the conditional probability of enrolling in insurance given a set of observed covariates as defined by Rosenbaum and Rubin [40]. The propensity score estimation takes advantage of the covariates available in the KDHS and reduces bias due to differences in observed covariates thus balancing the covariates in the insured and uninsured groups. After using the logit model in estimating the propensity scores and achieving balance of the propensity score between the insured and uninsured, the goal was to estimate the Average Treatment Effects (ATE) or population effects of enrollment in insurance. ATE can be determined as the difference in average outcomes for insured and uninsured and can be written as shown in eq. 2:

2 $$ ATE={\sum}_{i=0}^n\left({y}_{1\mathrm{i}}-{y}_{0\mathrm{i}}\right) $$

where *n* = the total number of pregnant women; *y*_1i_ is outcomes for the insured; and *y*_0i_ is outcomes for the uninsured. However, we cannot estimate eq. (2) as we cannot observe both *y*_1i_ and *y*_oi_ (counterfactuals/potential outcomes) for every pregnant woman. And given that our study is observational, it is likely that the outcomes of interest (institutional delivery and access to skilled birth attendant) are dependent on treatment (insurance enrollment) leading to biased ATE. We therefore use the propensity scores for estimation of the causal association of enrollment in health insurance. Specifically we estimate and report the ‘*Average Treatment Effect on the Treated*’ (ATT) i.e. the average response to treatment (insurance) for those pregnant women that enrolled in or were enrolled in health insurance. From the ATE equation above (equation 2), we estimate the ATT equation below:

3 $$ ATT= E\left({y}_{1 i}-{y}_{0 i}\left| X, Z=1\right.\right) $$

where *X* is a set of covariates to condition on and *Z* is the treatment (enrollment in health insurance). The ATT estimation is based on the following assumptions [39–43]:

1. *Stable unit treatment value assumption (SUTVA*): the treatment applied to one entity does not affect the outcome of any other (i.e. no interference among pregnant women)
2. *Positivity*: requires that there be a non-zero probability of receiving every level of treatment (insurance enrollment) for the combination of values of exposure and covariates that occur among entities in the population (pregnant women). The positivity assumption can be made when each homogeneous entity can be assigned to the treatment (insured) or non-treatment (uninsured) group.
3. *Unconfoundedness*: the treatment assignment mechanism is said to be unconfounded if the treatment status *T*_*i*_is conditionally independent of the potential outcomes, given a set of covariates *x*_*i*_. This is represented as shown in eq. 4 below:

4 $$ {T}_{\mathrm{i}}\coprod {y}_{0\mathrm{i}},{y}_{1\mathrm{i}}\left|{x}_{\mathrm{i}}\right. $$

These assumptions allowed for the construction of matched insurance samples based on the balancing score – the propensity score [40] and estimation of causal association of enrollment in health insurance by stratification, kernel, and nearest neighbor matching. We also conducted inverse probability weighting (IPW). Given that this study is an observational cross-sectional study with a single treatment variable, as discussed by Bender and Lange 2001, multiple test adjustments were not performed [44]. We used sampling weights in all analysis to account for the complex sampling strategy in the KDHS discussed above, and all statistical analyses were implemented in Stata 13.

### Heterogeneous effect

The average effects estimated from the linear, logistic, and propensity score methods may be heterogeneous for those with and without insurance. We addressed the potential for impact heterogeneity by further stratifying the analysis based on socio-economic status (SES). The SES index is a binary variable based on having electricity at home and current employment status. Because about 75% of the study sample lives in rural areas, having electricity and or working are good proxies for higher SES status.

## Results

**Fig. 1:**
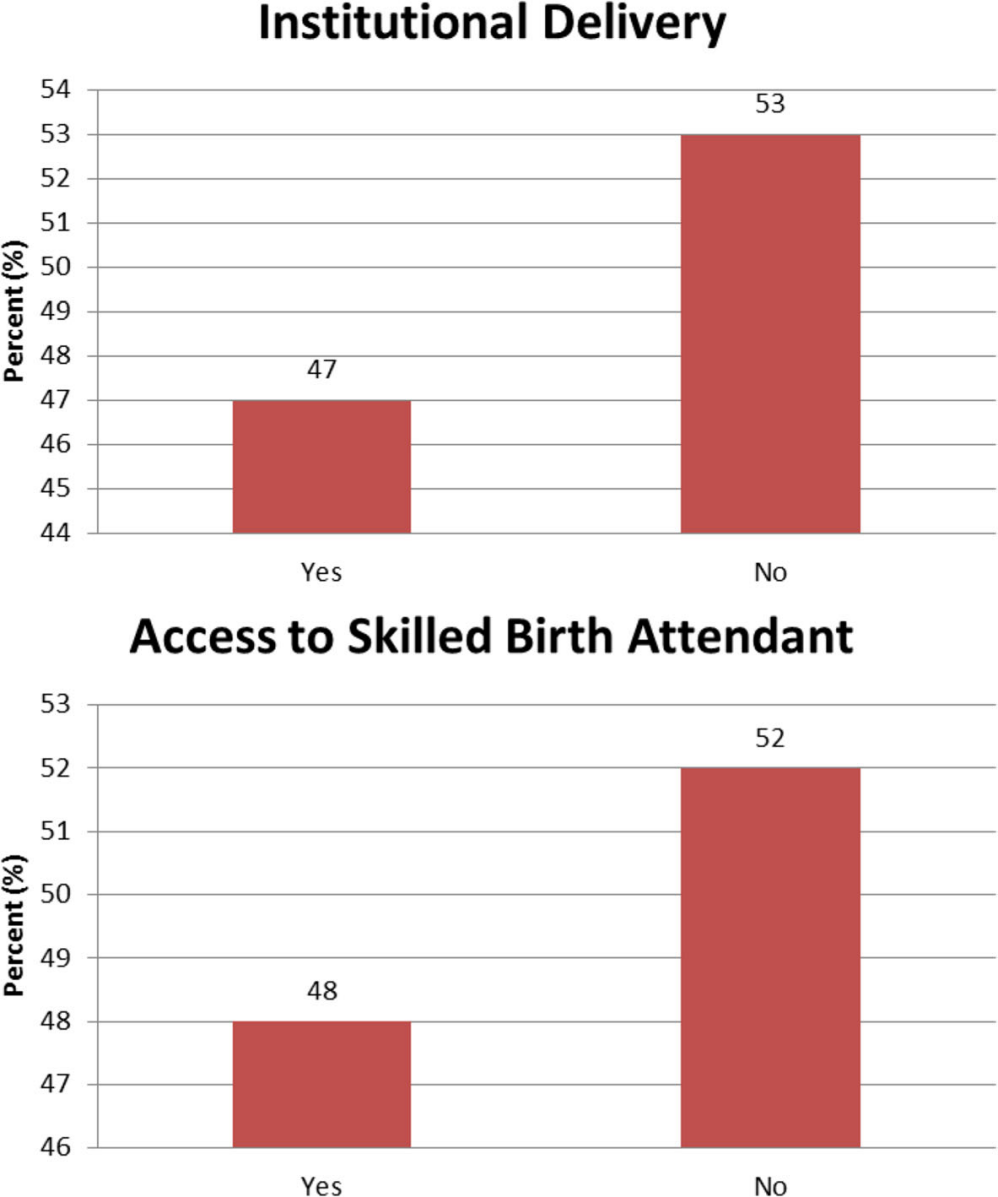
Primary Outcomes based on DHS Kenya 2008–09 Data

**Fig. 2:**
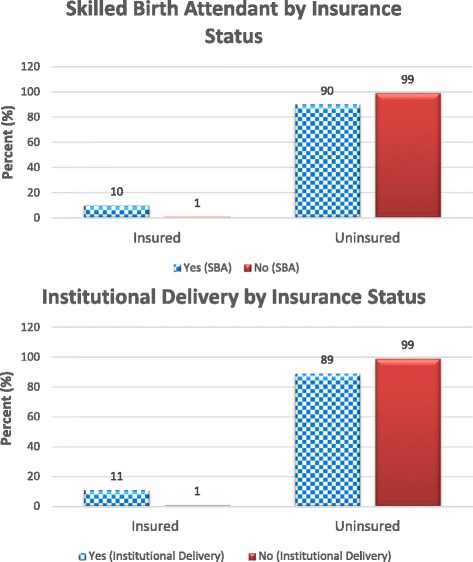
Outcomes by Insurance Status

From the 2008–09 KDHS only 7% of the women are enrolled in health insurance. As shown in Fig. 1, 47 and 48% of mothers deliver at institutions and have access to skilled birth attendants respectively. In Fig. 2, 11% of those delivering at institutions are insured compared to 1% of those not delivery at institutions. The scenario is similar for access to skilled birth attendant where 10% of those having access to SBA have insurance and 1% do not. Table 1 presents the means of the sociodemographic characteristics of the insured and uninsured. On average, the insured are older, are more educated, are more likely to be currently working, live in urban areas, be married and have electricity. The insured have fewer children and smaller households. Also the Kikuyu and Kalenjin are more likely to have insurance. Table 2 shows the balance of the mean propensity score by blocks and Additional file 1: Table A1 in the appendix shows the test of the balancing property for variables by blocks. In all nine blocks the mean propensity score is not different for the treatment and controls. The test of the balance of the propensity score divides the propensity scores into blocks and tests for balance of the propensity score between those with insurance and those without insurance within the stratified blocks.

**Table 1:** Sociodemographic Characteristics of Pregnant Women in Kenya (2008–09 DHS)

Mean Values	Overall Sample (*N* = 4082)	Insured (*N* = 231)	Uninsured (*N* = 3851)
Age (years)	28.49	31.72	28.17
Total # of Children	2.68	2.08	2.71
# of Household Members	5.52	4.71	5.54
Years of Education	7.85	12.8	7.003
Currently Working	56.6	74.5	51.57
Taken a HIV Test	58.45	75.17	56.9
Marital Status:			
Never Married	31.2	26.15	30.37
Married	54.22	63.74	54.81
Living together	4.14	4.31	4.25
Widowed	4.37	1.82	4.34
Divorced	1.27	1.00	1.43
Separated	4.8	2.98	4.8
Ethnicity:			
Kalenjin	13.21	20.59	14.86
Kamba	10.93	10.43	7.69
Kikuyu	19.44	23.01	17.41
Kisii	6.86	7.29	5.14
Luhya	16.26	11.92	15.23
Luo	13.00	13.41	13.16
Meru/Embu	4.92	3.81	4.39
Mijikenda/Swahili	5.09	2.82	8.93
Somali	2.84	1.16	8.57
Taita/Taveta	0.94	2.65	1.38
Maasai	1.34	1.00	1.52
Embu	1.42	1.66	1.72
Other, unspecified	3.75	0.33	0.00
Living in Urban Areas	25.44	65.40	28.32
Electricity	22.81	68.38	20.75

Notes: The table shows the characteristics of women in the study sample that were surveyed as part of the 2008–09 nationally representative survey the Demographic and Health Survey (DHS) in Kenya. 8444 women aged 15–49 years were selected from a sampling frame of 400 sampling units across eight provinces of Kenya with rural and urban residential stratification. For this study a total of 4082 women who reported being pregnant and giving birth are analyzed. Values are percentages, unless otherwise noted

**Table 2:** Test of the Balance of Mean Propensity Score by Blocks

Block	Insured	Uninsured	*P*-Value
Block 1	0.01	0.01	0.9353
Block 2	0.02	0.02	0.9403
Block 3	0.04	0.03	0.4356
Block 4	0.08	0.07	0.0353
Block 5	0.13	0.12	0.2706
Block 6	0.18	0.17	0.1058
Block 7	0.31	0.28	0.0132
Block 8	0.49	0.48	0.5733
Block 9	0.69	0.67	0.3561
*N* = 3689	231	3458	

Note: The above table reports the test of balance of the mean propensity score between the treated and controls by blocks. The results are from implementing *“pscore.ado”* program in Stata. The *P*-values are based on a two-sample t-test with equal variance. The *pscore* command fits a logit (probit is the default) model with a starting specification of linear terms without interactions or higher order terms. If balance in not achieved in a block, the sample in the block is split into equally spaced intervals, with higher order terms and interactions included, and the average propensity score of the treated and controls is re-tested till balance is achieved

Table 3 shows results from the linear and logistic regression models indicating that having insurance is associated with increased likelihood of institutional delivery and access to SBA. For example in the unadjusted models, mothers with insurance are 44 and 43 percentage points more likely to deliver at an institution and have access to a skilled birth attendant respectively. While in the adjusted models there is attenuation of association, the coefficients are still large and statistically significant at the 1% level. These significant effects also hold in the propensity score models as shown in Table 4. The IPW estimates show that mothers with insurance are 23 percentage points more likely to deliver at an institution and 20 percentages points more likely access to SBA. The numerical differences in the association estimates in Table 4 reflect how the different matching strategies – stratified; kernel; & nearest neighbor matching – were implemented in forming the matched sets of treated (insured) and untreated (uninsured) subjects who share the same propensity score [45].

**Table 3:** Linear and Logistic Regression Estimates of the Association of Insurance with Institutional Delivery & Skilled Birth Attendant

	Institutional Delivery				Skilled Birth Attendant			
	1 Linear Unadjusted	2 Linear Adjusted	3 Logistic Unadjusted	4 Logistic Adjusted	5 Linear Unadjusted	6 Linear Adjusted	7 Logistic Unadjusted	8 Logistic Adjusted
Insurance	0.439*** (0.023)	0.129*** (0.026)	0.439*** (0.023)	0.216*** (0.038)	0.433*** (0.0213)	0.146*** (0.025)	0.433*** (0.0213)	0.251*** (0.037)
N	4082	4082	4082	4082	4.082	4082	4082	4082
Constant	0.444*** (0.008)	0.115** (0.0502)			0.467*** (0.008)	0.164*** (0.051)		
R Squared	0.0413	0.3253	0.033	0.281	0.04	0.30	0.033	0.258

Notes: In the table above models 1 & 5 are unadjusted linear models and 3 & 7 are unadjusted logistic models. Models 2 & 6 are linear models with controls and 4 & 8 are logistic with controls. The vector of controls includes age, household characteristics, education, pregnancy history, HIV test, and urban residence. Reported for models 3, 4, 7 & 8 are average marginal effects and the R squared is Pseudo R2. In parentheses are robust Std Errors. Significance levels: ***p < 0.01, **p < 0.05, *p < 0.1

**Table 4:** Estimates of the Association of Insurance with Institutional Delivery & Skilled Birth Attendant based on Propensity Score Methods

	Institutional Delivery				Skilled Birth Attendant			
	1 Stratified	2 Kernel	3 Nearest Neighbor	4 IPW	5 Stratified	6 Kernel	7 Nearest Neighbor	8 IPW
Coefficients (ATT)	0.120***	0.180***	0.110**	0.231***	0.130***	0.187***	0.123***	0.200**
Std. Errors	0.023	0.023	0.035	0.034	0.024	0.024	0.032	0.092
T/Z- Statistic	5.151	7.673	2.502	6.77	5.322	7.913	3.830	2.17
N								
Analytic Sample	3689	3689	3689	3689	3689	3689	3689	3689
Treatment	231	231	231		231	231	231	
Control	3458	3458	188		3458	3458	188	

Notes: ATT = Average Treatment Effect on the Treated. In the above table model 1 & 5 is stratified matching; model 2 & 6 is kernel matching); model 3 & 7 is nearest neighbor matching - random draw version; and model 4 & 8 is inverse probability weighting (IPW) by logistic regression. The standard errors are bootstrapped standard errors (100 reps). Significance levels: ***p < 0.01, **p < 0.05, *p < 0.1

The heterogeneous effect estimates based on socioeconomic status in Table 5 show that both those of lower and higher SES benefit from having insurance. However, those of lower SES seem to benefit more from enrollment in insurance compared to those mothers of higher SES. For both institutional delivery and access to SBA the average ATT and IPW effect estimates of insurance for those of low SES is 23 percentage points, and 6 percentage points for those of higher SES.

**Table 5:** Heterogeneous Effect Estimates – Socio-Economic Status

	SES INDEX					
	HIGH			LOW		
	1 Kernel	2 Nearest Neighbor	3 IPW	4 Kernel	5 Nearest Neighbor	6 IPW
Panel A: Institutional Delivery						
Coefficients (ATT)	0.052*	0.065*	0.090**	0.293***	0.311***	0.193*
Stand Errors	0.025	0.039	0.032	0.076	0.109	0.120
T- Statistic	2.059	1.662	2.80	3.873	3.318	1.66
N						
Analytic Sample	661	661	661	2691	2691	2691
Treatment	139	139		53	53	
Control	519	101		2271	55	
Panel B: Skilled Birth Attendant						
Coefficients (ATT)	0.068***	0.086**	0.099***	0.296***	0.330**	0.186*
Stand Errors	0.022	0.029	0.030	0.061	0.114	0.122
T- Statistic	3.105	2.303	3.31	4.818	2.890	2.06
N						
Analytic Sample	661	661	661	2691	2691	2691
Treatment	139	139		53	53	
Control	519	101		2271	55	

Notes: ATT = Average Treatment Effect on the Treated. In the above table model 1 & 4 is Kernel matching; model 2 & 5 is Nearest Neighbor matching - random draw version; and model 3 & 6 is Inverse Probability Weighting (IPW) by logistic regression. The SES index is a binary variable based on having electricity at home and current employment status. The standard errors are bootstrapped standard errors. Significance levels: ***p < 0.01, **p < 0.05, *p < 0.1

## Discussion

This study assessed the association of insurance enrollment on institutional delivery and access to skilled birth attendants in Kenya. The findings of this study suggest that enrollment in insurance improves access to institutional delivery and skilled birth attendants (SBA) for pregnant women and is consistent with the literature on this topic. For example, Wang et al. did a similar study on the impact of health insurance on maternal healthcare utilization using DHS data and propensity score matching [46]. Nevertheless, their study looked at three countries with high levels of insurance coverage (Ghana, Indonesia, and Rwanda) unlike Kenya. And of the four outcomes analyzed, three outcomes based on antenatal care visits were different from those analyzed in this paper- only institutional delivery was similar. Also, across the three countries, the Wang et al. paper found significant and positive results on at least two of the four outcome measures [46]. The positive association of insurance found in our study on Kenya as well as the study by Wang et al. complement those reported in the systematic review by Comfort et al. that indicates that there is relatively constant evidence that health insurance in positively correlated with use of maternal health services [47]. As such, this paper adds to the literature by not only showing that insurance is critical and beneficial for maternal health outcomes, but it also highlights the value of insurance in countries that have low enrollment rates especially among those of lower socioeconomic status like Kenya. These findings are thus important given the ongoing healthcare financing reforms not only in Kenya but also in other similar sub-Saharan Africa countries [17, 35].

And although NHIF is one of the oldest social health insurance programs in Africa, health insurance reform and evaluation is still at an early stage in Kenya. This study is, to the best of our knowledge, one of the few evaluations aiming to provide evidence for policy formulation and implementation in Kenya. The ongoing health financing reforms are focused on NHIF as the primary vehicle for health care financing. This is despite reforms taking place without a local evidence base on the impact of NHIF on various outcomes. Thus, given the higher rates of maternal and child morbidity and mortality in Kenya, the government should actively promote the use of health insurance to facilitate access to needed and critical care as this may be a much more efficient, and sustainable way of financing care than user fees. This is especially the case as our study shows that mothers of lower SES stand to benefit the most from enrolling in insurance. Such findings are policy relevant and give impetus for the ongoing social health insurance reforms in Kenya. In addition, the protective association of insurance on those of lower SES supports the push by social scientists and policy makers for SHI as a viable option to improve population access to reproductive health services, while improving health outcomes for disenfranchised populations [17, 48]. These findings should thus be of interest to healthcare policy makers and stakeholders in Kenya.

Nonetheless, we should take these findings with caution given the non-random enrollment into insurance and the observational and cross-sectional nature of the data as this study is limited in causal estimation compared to an experimental study design. However, given the extensive nationally representative data and covariates used in the analysis, the study was able to mimic a quasi-experimental design using regression adjustment, propensity score adjustment and inverse probability weighting, and thus make empirical estimations of the relationship between insurance and healthcare utilization outcomes for pregnant women. Moreover, the data does not allow for the estimation of other utilization parameters such as costs, out of pocket payments or the distribution of obstetric healthcare workers. Further research is thus needed and should focus on experimental designs, particularly of innovations aimed at improving the current system, as well as collection of longitudinal data to support causal inference analyses overtime. And studies on the factors that predict insurance enrollment should be conducted so that these factors can be targeted to improve insurance coverage and enrollment.

## Conclusion

Pregnant women enrolled in health insurance have greater access to institutional delivery and skilled birth attendants in comparison to pregnant women not enrolled in health insurance. Of critical importance, enrollment in health insurance is beneficial for pregnant women of lower socio-economic status in Kenya. Despite the limitations of the cross-sectional data utilized in this analysis, the findings presented show that as healthcare financing reforms are instituted in Kenya and other developing countries with similar settings, health insurance enrollment initiatives should be made accessible to pregnant women especially those of lower socio-economic status.

## Additional file

Additional file 1: Figure A1. Graphs of delivery services user fees by facility type and managing authority for 2004 and 2010. **Fig. A2.** Graphs of charging for normal delivery by facility type and managing authority for 2004 and 2010. **Table A1.** Results of the testing of the balancing property for variable used in the estimation of the propensity score by blocks. (DOCX 120 kb)

## Abbreviations

DHS: Demographic and Health Survey
IFC: International Finance Cooperation
IPW: Inverse Probability Weighting
KDHS: Kenya Demographic and Health Survey
MDG: Millennium Development Goals
MMR: Maternal Mortality Rate
NGO: Non-Governmental Organizations
NHIF: National Health Insurance Fund
OOP: Out-of-Pocket
SBA: Skilled Birth Attendants
SES: Socio-Economic Status
SHI: Social Health Insurance
SPA: Service Provision Assessment
UHC: Universal Health Coverage
WHO: World Health Organization
