# Supplementary material for: The Association of Health Insurance with institutional delivery and access to skilled birth attendants: evidence from the Kenya Demographic and health survey 2008–09
Source: BMC Health Serv Res. 2017 Jul 3;17:454. doi: 10.1186/s12913-017-2397-7 (PMC5496351; doi:10.1186/s12913-017-2397-7)
Supplement: Additional file 1: Figure A1. — Graphs of delivery services user fees by facility type and managing authority for 2004 and 2010. Fig. A2. Graphs of charging for normal delivery by facility type and managing authority for 2004 and 2010. Table A1. Results of the testing of the balancing property for variable used in the estimation of the propensity score by blocks. (DOCX 120 kb) [file 12913_2017_2397_MOESM1_ESM.docx]

**Appendix:**

**Contents:**

Figure A.1 Graphs of Delivery Services User Fees by Facility & Managing Authority ……………………… 2

Figure A.2 Graphs of Charging for Normal Delivery by Facility & Managing Authority …………………… 3

Table A.1 Test of Balancing Property for Variables by Blocks……………………………………………………..... 4

Figure A.1: Graphs of Delivery Services User Fees by Facility & Managing Authority


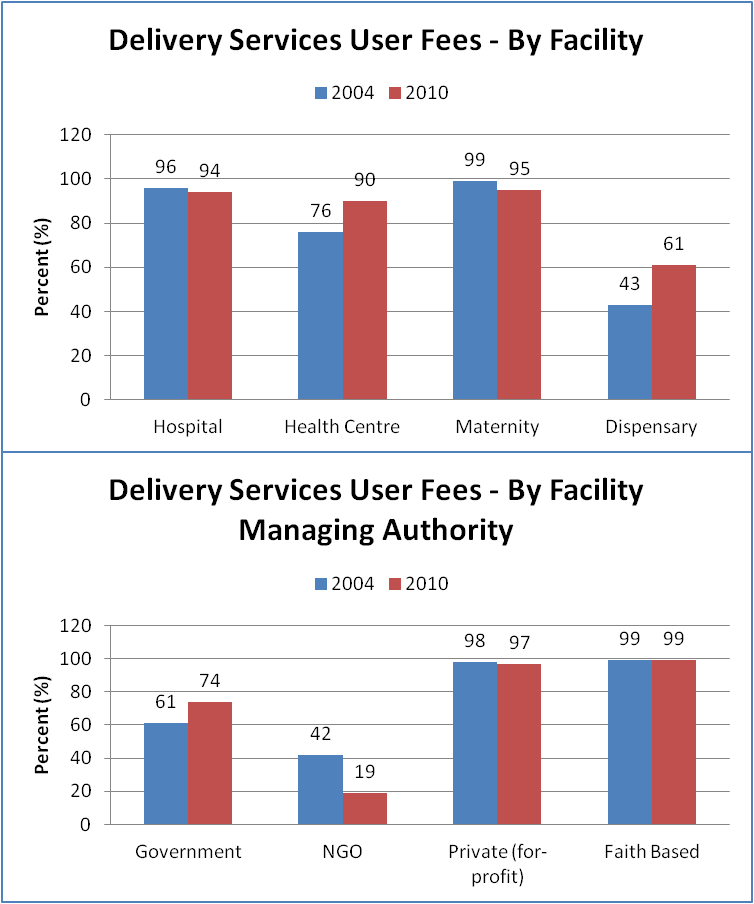


Note: The above graphs were generated using the 2004 and 2010 Kenya Service Provision Assessment Surveys.

KSPA provide national and sub-national information on the availability and quality of services from a

representative sample of health facilities. These facilities include hospitals, health centers, dispensaries,

maternities, clinics, and VCT centers. The 2004 and 2010 samples were drawn from 440 and 703 health facilities respectively.

Figure A.2: Graphs of Charging for Normal Delivery by Facility & Managing Authority


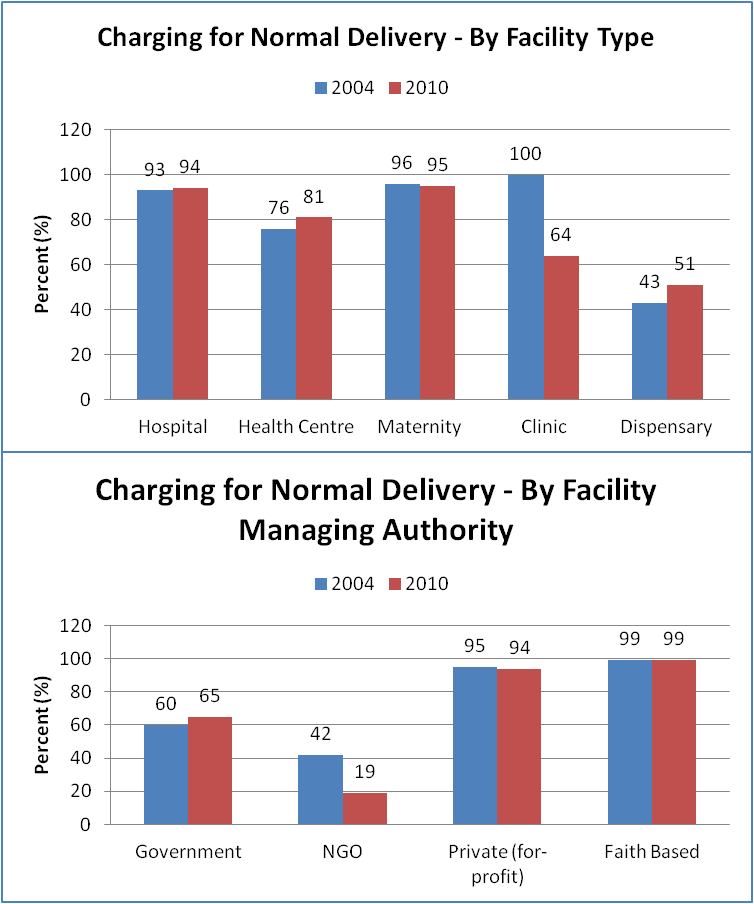


Note: The above graphs were generated using the 2004 and 2010 Kenya Service Provision Assessment Surveys.

KSPA provide national and sub-national information on the availability and quality of services from a

representative sample of health facilities. These facilities include hospitals, health centers, dispensaries,

maternities, clinics, and VCT centers. The 2004 and 2010 samples were drawn from 440 and 703 health facilities respectively.

| Table A.1: Test of Balancing Property for Variables by Blocks | | | |
| --- | --- | --- | --- |
| **Block 1** | | | |
| **Variable** | **Mean for Insured (n=8)** | **Mean for Uninsured (n=1107)** | **P-Value** |
| Age (yrs.) | 28.5 | 26.2 | 0.3653 |
| Education (%) | 0.75 | 0.72 | 0.8506 |
| Urban (%) | 0.25 | 0.18 | 0.6070 |
| Electricity (%) | 0.13 | 0.03 | 0.0776 |
| Currently Working (%) | 0.13 | 0.24 | 0.4380 |
| Information (%) | 0.00 | 0.01 | 0.7492 |
| HIV Test (%) | 0.75 | 0.72 | 0.8592 |
| Cooking Fuel (%) | 0.25 | 0.18 | 0.5910 |
| Total No. of Children | 4.88 | 3.62 | 0.1649 |
| Total No. of Household | 6.13 | 6.00 | 0.8884 |
|  |  |  |  |
| **Block 2** | | | |
| **Variable** | **Mean for Insured (n=21)** | **Mean for Uninsured (n=824)** | **P-Value** |
| Age (yrs.) | 27.14 | 27.32 | 0.9049 |
| Education (%) | 0.95 | 0.96 | 0.8854 |
| Urban (%) | 0.14 | 0.16 | 0.7976 |
| Electricity (%) | 0.10 | 0.02 | 0.0175 |
| Currently Working (%) | 0.81 | 0.73 | 0.4202 |
| Information (%) | 0.00 | 0.08 | 0.1920 |
| HIV Test (%) | 0.81 | 0.79 | 0.8187 |
| Cooking Fuel (%) | 0.19 | 0.20 | 0.8909 |
| Total No. of Children | 3.91 | 3.64 | 0.6196 |
| Total No. of Household | 5.76 | 5.80 | 0.9445 |

Note: The above table shows the result of testing the balancing property for variables used in estimation of the propensity score by implementing *“pscore.ado”* program in Stata. The P-values are based on a two-sample t-test with equal variance. The *pscore* command fits a logit (probit is the default) model with a starting specification of linear terms without interactions or higher order terms. If balance in not achieved in a block, the sample in the block is split into equally spaced intervals, with higher order terms and interactions included, and the average propensity score of the treated and controls is retested. Once all the blocks are balanced, the means of each covariate within each block are tested for balance i.e. the means of each variable do not differ between treated and control units.
